# The Role of Omega-3 Fatty Acids in Horses’ Nutrition—A Review

**DOI:** 10.3390/ani16111626

**Published:** 2026-05-27

**Authors:** Julia Bronś, Katarzyna Czyż, Anna Wyrostek, Jakub Smoliński, Wojciech Kruszyński, Ewa Sokoła-Wysoczańska, Karolina Dorobisz

**Affiliations:** 1 Student Research Club for Sheep and Fur Animal Breeding “FutrOwce”, Institute of Animal Breeding, Wroclaw University of Environmental and Life Sciences, ul. Chełmońskiego 38 c, 51-630 Wroclaw, Poland; 2 Institute of Animal Breeding, Wroclaw University of Environmental and Life Sciences, ul. Chełmońskiego 38 c, 51-630 Wroclaw, Poland; katarzyna.czyz@upwr.edu.pl (K.C.); anna.wyrostek@upwr.edu.pl (A.W.); jakub.smolinski@upwr.edu.pl (J.S.); 3 Department of Genetics, Wrocław University of Environmental and Life Sciences, ul. Kożuchowska 7, 51-631 Wroclaw, Poland; wojciech.kruszynski@upwr.edu.pl; 4 The Lumina Cordis Foundation, Szymanowskiego 2a, 51-609 Wroclaw, Poland; 5 Department of Otolaryngology, Head and Neck Surgery, Wrocław Medical University, 50-367 Wroclaw, Poland; karolina.dorobisz@umw.edu.pl

**Keywords:** mares, foals, stallions, fatty acids, omega-3

## Abstract

This narrative review presents examples of omega-3 fatty acids supplementation in horse nutrition. Fats are essential in equine nutrition, serving not only as an energy source but also as functional components influencing health. Among them, omega-3 polyunsaturated fatty acids (PUFAs), particularly EPA and DHA, play a key role in modulating inflammatory and metabolic processes. This review summarizes their effects on respiratory health, equine metabolic syndrome (EMS), musculoskeletal function, reproduction, and early development. Omega-3 supplementation has been shown to support airway inflammation in horses, especially when combined with low-dust management practices. In EMS, these fatty acids support insulin regulation and reduce systemic inflammation, although they are not curative. In the musculoskeletal system, omega-3s help limit inflammation and cartilage degradation. Their reproductive benefits include improved semen quality in stallions and enhanced fatty acid profiles in mare’s milk, supporting foal development. The proposed mechanisms of omega-3 fatty acids’ effect are also provided. Overall, omega-3 fatty acids are a valuable supplement in equine functional nutrition and disease management.

## Simple Summary

Fats are an important part of a horse’s diet, not only as a source of energy but also as an additive supporting disease treatment and for maintaining a general good condition. In particular, omega-3 fatty acids—especially EPA and DHA—have been shown to support many key functions in the body. This article reviews how these supplements affect horses’ breathing, metabolism, joints, reproduction, and early development. The research shows that omega-3s can help reduce inflammation in the airways, especially when horses are also kept in low-dust environments. They may also support better control of blood sugar in horses with metabolic problems, although they are not a cure. In joints, omega-3s can help reduce inflammation and protect cartilage. They may also improve semen quality in stallions and enrich mare’s milk, which supports the healthy growth of foals. Overall, omega-3 fatty acids can be a helpful addition to a horse’s diet, supporting their health and wellbeing when combined with proper care and feeding practices.

## 1. Introduction

**Figure 1:**
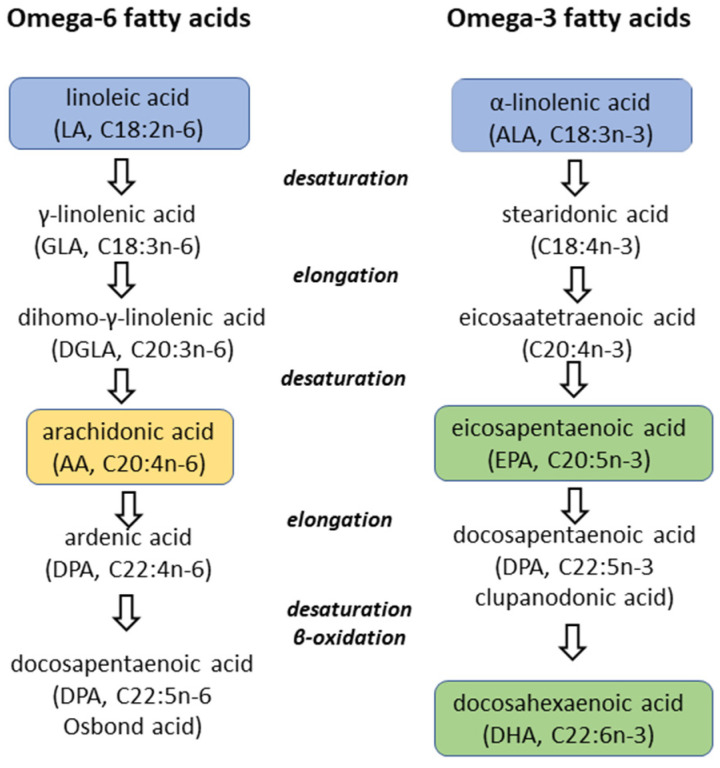
Metabolic pathway of omega-6 and omega-3 fatty acids (own elaboration based on [1,2,4,6]).

Fats are an important part of the diet of humans and animals because they provide energy and play an important role in the proper functioning of the body. Essential fatty acids (EFAs), which belong to the group of polyunsaturated fatty acids, are particularly important. They are essential for maintaining physiological homeostasis, supporting optimal growth and development, and preserving psychophysical equilibrium. The body cannot produce them on its own, so they must be supplied through diet or supplements. Omega-3 acids can be found in vegetable oils, such as flaxseed, canola, soybean, and hemp oil; nuts, such as walnuts; and in seeds, dairy products, eggs, algae and cold-water fish; omega-6 acids are mainly derived from animal fat, but also from plant sources, such as corn, soybean, and sunflower oil [1,2]. From a structural standpoint, the biochemical distinctions between omega-3 and omega-6 fatty acids appear marginal, yet they elicit vastly different physiological responses [3]. The main differences are the location of the first double bond; in omega-3 FAs it is located on the third, while in omega-6 FAs it is on the sixth atom of carbon at the methyl terminus of the chain [4]. The most important EFAs include two acids: α-linolenic acid (ALA, 18:3n-3) from the omega-3 family and linoleic acid (LA, C18:2n-6c), which belongs to the omega-6 group [5]. ALA is used to produce other omega-3 fatty acids, including eicosapentaenoic acid (EPA, 20:5n-3) and docosahexaenoic acid (DHA, 22:6n-3), while LA is used to synthesize other omega-6 fatty acids. These compounds can be converted into long-chain fatty acids through desaturation and elongation processes [4,6]. The metabolic pathway of omega-3 and omega-6 fatty acids is presented in Figure 1.

In the case of omega-6 fatty acids, linoleic acid (LA) synthesizes γ-linolenic acid (GLA), which further synthesizes dihomo-γ-linolenic acid (DGLA) and arachidonic acid (AA), the major product of omega-6 family [4,5]. The main substrate in omega-3 fatty acids synthesis is α-linolenic acid (ALA), which is converted into stearidonic acid, eicosatetraenoic acid, and then into eicosapentaenoic acid (EPA). EPA is then transformed into docosapentaenoic acid (DPA), and the final product is docosahexaenoic acid (DHA) [1,4]. Omega-6 fatty acids, due to enzymatic metabolism processes, produce eicosanoids of the 2 and 4 series, which are characterized by pro-inflammatory properties, while omega-3 fatty acids form eicosanoids of the 3 and 5 series, exhibiting anti-inflammatory features [1,5,7,8]. The same enzymes, i.e., elongases and desaturases, are involved in the transformations of both acid groups, which is why omega-3 and omega-6 acids compete for access to elongases and desaturases, especially Δ6-desaturase. Too much LA in the diet can limit the conversion of ALA to EPA and DHA. Studies have shown that the efficiency of ALA conversion to EPA is about 6–8%, while to DHA it is only about 4% [7,8,9,10].

In recent years, the research on omega fatty acids in horses has evolved from analyzing them as an additional source of energy to their role in functional nutrition. The latest literature emphasizes not only the presence of fat in the diet, but also specific long-chain fatty acids (EPA and DHA) and their direct impact on the expression of inflammatory genes. This paper reviews the current literature on supplementation with various omega-3 fatty acid sources in equine nutrition, specifically evaluating their efficacy for mitigating specific diseases and physiological conditions. Furthermore, this review identifies gaps in the current literature to provide strategic directions for future research.

## 2. Materials and Methods

This paper is constructed as a narrative review presenting attempts to use omega-3 fatty acids in equine nutrition, focusing on their possible supplementation in cases of the most common diseases in horses, and in relation to reproduction and foal development.

A literature search was performed using PubMed and Google Scholar databases. Combinations of the following keywords were used: omega-3 fatty acids, horse nutrition, horse supplementation, airway inflammatory disease, equine metabolic syndrome, laminitis, joints, joint inflammation, mare milk, foal development, horse reproduction, and stallion semen.

First, the titles and abstract were screened to verify their relevance, then the full texts were evaluated for their suitability. Lists of references of selected articles were also screened to find additional relevant papers.

The review included studies that presented data on horses’ diet supplementation with omega-3 fatty acids, and their effects on airway inflammatory diseases, equine metabolic syndrome, joints and musculoskeletal system of horses, foal development, and stallion reproduction. Studies were excluded if they were not directly related to the topic. Some additional studies, not presenting omega-3 acids in horse nutrition, were also included when they were related to the topic discussed and could support the observations and findings from the other studies.

## 3. Airway Inflammatory Diseases

The most significant chronic lower airway inflammatory diseases in horses include recurrent airway obstruction (RAO) and inflammatory airway disease (IAD). According to Nogradi et al. [11], their prevalence in the Northern Hemisphere is 14 and 20%, respectively. In both cases, horses show symptoms related to chronic airway inflammation, like coughing and excessive airway mucous; however, only horses with RAO demonstrate an increased respiratory effort at rest and exercise intolerance, while in the case of IAD the clinical symptoms are less severe and include impaired performance and occasional coughing with normal breathing at rest [11,12]. Moreover, RAO usually affects horses older than 7 years, while IAD is not related to age. According to the Consensus Statements of the American College of Veterinary Internal Medicine (ACVIM) [12], both these conditions are commonly referred to as equine asthma syndrome.

Nogradi et al. [11] conducted a study in which they examined the effectiveness of feed supplement rich in omega-3 PUFAs, in addition to a low-dust diet, in horses with chronic airway disease (both RAO and IAD) compared to healthy horses. The authors focused on the changes in the clinical score, pulmonary function, and BALF (bronchoalveolar lavage fluid) cytology parameters. Supplementation with polyunsaturated fatty acids (PUFAs) led to a marked increase in plasma docosahexaenoic acid (DHA) levels, which peaked after 4 weeks of use. Although overall clinical improvement was observed in all animals participating in the study, the group receiving PUFAs achieved significantly better results (60% improvement in cough score, 48% reduction in respiratory effort, and neutrophil count decrease from 23 to 9%) than the control group (33% improvement in cough score, 27% reduction in respiratory effort, and increase in neutrophil count in BALF from 11 to 17%). The most important difference, apart from the scale of improvement itself, was the effect on lung cytology (BALF). While the horses receiving PUFAs showed a more than twofold decrease in their neutrophil count (indicating inflammation suppression), the inflammatory parameters worsened in the placebo group. However, it should be remembered that the elimination of dusty environment was an important element of this study, and lack of it would have probably, to some degree, reduced the benefits of omega-3 supplementation; thus, supplementation acted as a supportive tool. Moreover, the study suggested that a limited amount of DHA can be absorbed and integrated into the plasma to achieve an anti-inflammatory effect, and higher doses may not result in additional health benefits [11]. The authors concluded that the clinical improvement observed in the study could have resulted from the fact that the omega-3 fatty acids from the supplement shifted the enzymatic conversion of arachidonic acid towards less inflammatory compounds [11,13]. They also suggested that an analysis of other oxidative stress markers would be useful for further interpretation of the results obtained [11].

The study conducted by Khol-Parisini et al. [14] investigated whether supplementing the diet with different types of fat could alleviate the symptoms of recurrent airway obstruction in horses. It compared sunflower oil, which is rich in omega-6 fatty acids, mainly linoleic, with seal blubber oil, a source of long-chain omega-3 polyunsaturated fatty acids (PUFAs). The study included nine horses suffering from RAO, who received 320 mg/kg body weight of supplement for 10 weeks. The authors examined the fatty acid ratios in their plasma and leukocyte membranes, performed lung function tests, and analyzed the cellular composition of the pulmonary epithelial lining fluid (PELF). The study demonstrated that the supplemental fatty acids were successfully incorporated into both the plasma and leukocyte cell membranes. Seal blubber oil supplementation significantly reduced the omega-6 to omega-3 ratio in both the plasma and leukocyte phospholipids, and also led to a significant reduction in the total number of leukocytes in the lungs (PELF), suggesting a decrease in local airway inflammation. However, despite the reduction in inflammatory cells, there were no significant improvements in the clinical respiratory scores or pulmonary function (e.g., airway resistance or lung compliance) [14]. The authors suggested that while omega-3 PUFAs can reduce the cellular component of inflammation (the number of white blood cells), they may not be sufficient to reverse the mechanical changes (bronchospasms and structural remodeling) already present in the lungs of horses with chronic RAO. This explains why the cells improved but the clinical signs (breathing) did not. The study confirmed that seal blubber oil (omega-3) can successfully modify the fatty acid profile of immune cells and reduce the cellular inflammatory response in the lungs of horses with RAO. However, as a standalone treatment, it may not be enough to improve the physical ease of breathing in horses with advanced airway obstruction [14].

In turn, Olave et al. [15] conducted a randomized clinical trial aimed at examining how switching from dry hay to low-dust forage (steamed hay or haylage) affects dust exposure and airway health in Thoroughbred racehorses. Although this was not a nutritional study, it is worth mentioning because it highlighted the importance of environmental dust on horses’ respiratory systems; furthermore, it showed the levels of omega-3 fatty acids in the blood in relation to the horses’ exposure to dust. The study included BALF cytology, plasma lipid profile and plasma SPM (specialized pro-resolving mediators) concentrations measured at the beginning and end of 6-week research period. The authors observed that feeding haylage or steamed hay reduced respirable dust exposure by up to 67% compared to dry hay. Steamed hay and haylage were associated with lower tracheal mucus scores and lower neutrophil counts in the airways. The study also monitored plasma omega-3 levels, noting that most racehorses on standard grain/hay diets are deficient in these fatty acids, which may exacerbate their inflammatory response to dust. The study demonstrated higher ratios of plasma EPA to AA in horses fed with haylage, and no differences in the case of other PUFAs concentrations. The authors suggested that steaming hay or using haylage physically binds dust particles and fungal spores to the forage, preventing them from being released into the breathing zone of horses. By reducing the dust load, there is a decrease in the oxidative stress placed on the pulmonary alveolar macrophages. The study also suggested that providing low-dust forage may create a baseline of health that allows for dietary omega-3 fatty acids to work more effectively by preventing constant re-exposure to inflammatory triggers [15].

## 4. Equine Metabolic Syndrome (EMS)

Equine metabolic syndrome (EMS) is a complex metabolic disorder characterized by an increased adiposity, insulin dysregulation, and laminitis or a predisposition to this condition [16,17]. EMS results from a complex interaction between genetic predisposition and environmental factors. Certain breeds, such as ponies, are genetically predisposed to EMS. They are often referred to as having a thrifty genotype, which originally allowed these animals to survive in harsh environments with sparse forage. Considering the environmental factors, it should be noted that modern management practices, including physical inactivity and diets high in non-structural carbohydrates (sugar and starch), are the primary environmental triggers [18]. Horses affected by EMS may exhibit low levels of systemic inflammation [19] or increased levels of tissue inflammation [20]. Further, inflammation leads to insulin dysregulation promotion, which may also result in a risk of laminitis development [16,17]. Moreover, obesity, especially regional adiposity (fat deposits in the neck or tail head), leads to the release of pro-inflammatory cytokines (adipokines) [16].

The occurrence of EMS has increased globally as the role of horses has shifted from a working animal to a leisure animal. There has also been a marked increase in equine obesity due to the lack of traditional land management knowledge and the high availability of nutrient-dense forage and supplementary feeds. While any horse can become obese, some are “high-genetic risk” individuals that develop insulin dysregulation even with moderate caloric intake. EMS is typically recognized in young to middle-aged horses (5–15 years), though it can persist or coexist with other conditions in older horses [21].

EMS is rarely an isolated condition; it is defined by several concurrent metabolic and physical states. The most critical accompanying state is hyperinsulinemia-associated laminitis (HAL). High levels of insulin directly damage the laminae of the hoof, leading to pain and potential structural failure. Another problem is regional adiposity; horses often display a cresty neck, fat deposits behind the shoulder, or fat pads around the tail head, even if the rest of their body is not overtly obese [21]. Dyslipidemia, i.e., elevated levels of circulating triglycerides, and altered blood pressure (hypertension) are sometimes observed in EMS-affected horses. Affected horses may also show abnormal levels of hormones like leptin (which is often elevated) and adiponectin (which is often decreased). In older horses, EMS may coexist with Pituitary Pars Intermedia Dysfunction (Cushing’s disease), which further complicates insulin regulation [22,23].

Elzinga et al. [17] conducted a study aimed at determining if supplementing horses suffering from EMS with DHA-rich microalgae improves insulin sensitivity and reduces systemic inflammation. It was a randomized, blinded, placebo-controlled study using horses with EMS, who were fed either a DHA-rich microalgae supplement or a placebo for 46 days. The study demonstrated that DHA supplementation significantly decreased the insulin concentration on day 46. However, no treatment effect on insulin sensitivity was noted. There was also a reduction in inflammatory markers, especially tumor necrosis factor (TNF-α), alpha-interleukin (IL-6, IL-1β, and IL-10) and interferon-gamma (INF-γ) expression in the blood of DHA-supplemented horses compared to the beginning of the experiment, but no differences in relation to the control horses were noted. The supplemented horses showed an improved insulin response during a combined glucose–insulin test (CGIT) [17].

Another study aimed to compare the effects of different sources of omega-3 fatty acids, i.e., marine (fish oil pellet and algae) vs. plant (ground flaxseed), on insulin sensitivity in healthy horses [24]. The study involved 21 mares that were assigned to three groups, control, flax, or marine, with supplementation for 90 days. The study demonstrated that while flaxseed increased certain omega-3 levels in the blood, it did not significantly improve insulin sensitivity. In turn, the study by Otabachian et al. [25] conducted on mares demonstrated that supplementation with omega-3 PUFAs caused increased insulin sensitivity. The marine supplement was much more effective at increasing the concentrations of long-chain fatty acids (DHA and EPA) in the plasma. The authors also noted that some breeds of horses may be more predisposed to insulin resistance [24].

The study by Loos et al. [26] evaluated a specific supplement’s ability to protect horses with insulin dysregulation (ID) when they were fed high-sugar/starch diets. While the study focused on a multi-ingredient nutraceutical, the fatty acid component, i.e., long-chain omega-3 polyunsaturated fatty acids (n-3 PUFAs), played a central role in the observed metabolic improvements. The nutraceutical used in the study contained a significant amount of DHA (docosahexaenoic acid) and EPA (eicosapentaenoic acid) derived from marine sources (microalgae and fish oil). In horses with a history of ID, the supplementation helped to maintain higher insulin sensitivity when the horses were challenged with a high-starch diet, compared to those receiving a placebo. No differences were observed in the basal glucose or insulin concentrations, but a lower glucose content at t = 45 min and a lower insulin concentration at t = 75 min were noted in the supplemented group. Also, the glucose clearance rates in the positive phase were higher in horses receiving the supplement. The study also demonstrated that omega-3 PUFA supplementation helped to regulate circulating triglycerides and non-esterified fatty acids (NEFAs) during the starch challenge [26].

Laminitis in the context of equine metabolic syndrome (EMS) is now specifically referred to as hyperinsulinemia-associated laminitis (HAL). Prolonged and excessive levels of insulin in the bloodstream (hyperinsulinemia) are directly responsible for the failure of the lamellae, the structural tissues that connect the hoof wall to the coffin bone. High insulin levels can cause inappropriate cell proliferation in the laminae or digital vasoconstriction, eventually leading to structural collapse and severe pain. Dietary fatty acids, particularly long-chain omega-3s (DHA and EPA), act as metabolic modifiers that help prevent the insulin spikes that cause laminitis. Omega-3 fatty acids are incorporated into cell membranes, increasing their fluidity. This physical change makes insulin receptors more efficient at binding insulin and moving glucose into cells (via GLUT4 transporters). When cells are more sensitive to insulin, the body does not need to produce as much of it to regulate blood sugar, thereby reducing the risk of the hyperinsulinemia that triggers laminitis. Omega-3 fatty acids compete with pro-inflammatory omega-6 acids to produce anti-inflammatory signaling molecules. They also inhibit the NF-κB pathway, a major inflammatory switch. By lowering systemic inflammation, fatty acids prevent inflammatory “noise” from blocking insulin receptors, further stabilizing a horse’s metabolic state [26,27,28]. It should be borne in mind, however, that the proposed mechanisms underlying the effects of omega-3 fatty acids are extrapolated from studies on other species and would need to be confirmed in studies on horses.

## 5. Joints and the Musculoskeletal System

Studies have suggested that omega-3 acids supplementation may positively affect the joints and musculoskeletal system in horses. The study conducted by Manhart et al. [29] aimed to examine mature horses with existing clinical arthritis (knee, fetlock, hock, or stifle joints). The horses in the experimental group received 15 g/d of EPA and 19.8 g/d of DHA for 90 days. Supplementation resulted in a significantly lower omega-6 to omega-3 FAs ratio in the horses’ diets, which was also reflected in the beneficial changes in this ratio found in their blood serum. The authors observed a decrease in the synovial fluid white blood cell counts in the supplemented horses compared to controls. Also, the plasma prostaglandin E2 and fibrinogen (an inflammatory marker) levels were significantly lower in the treated horses. The study suggested that long-term omega-3 supplementation may be effective as a tool supporting inflammation management in horses with chronic joint disease. In addition to reducing inflammation, it may also reduce joint pain level [29].

The study by Ross-Jones et al. [30] examined the effect of long-chain polyunsaturated fatty acids on experimentally induced synovitis in horses. Synovitis and joint effusion are suggested to be an inflammatory stage of osteoarthritis, which in turn is a condition characterized by degenerative changes in joint articular cartilage causing chronic pain and reduced mobility in horses. In the study, mares were fed 40 g/day of omega-3 PUFAs for 91 days. On day 91, an intra-articular injection of recombinant equine IL-1β (reIL-1β) was applied to stimulate joint inflammation. As a result of supplementation, the EPA and DHA concentrations increased significantly in both the serum and synovial fluid. Supplementation also contributed to a significant decrease in the expression of ADAMTS-4, an enzyme primarily responsible for breaking down the cartilage matrix (aggrecan). Unlike the natural arthritis study, this acute challenge model did not show a significant reduction in synovial PGE2. It may be suggested, based on this study, that long-chain polyunsaturated fatty acids may help to protect cartilage by modulating gene expression of degradative enzymes. However, the study used an acute arthritis model, which may have reduced beneficial effects of supplementation [30].

Leatherwood et al. [31] examined the effect of omega-3 acids supplementation on markers of inflammation in young horses in training. The authors provided the horses with a marine-origin supplement containing 15 g of EPA and 20 g of DHA during two phases of the experiment that differed in training intensity. They demonstrated an increase in plasma EPA and DHA concentrations as a result of supplementation, but it did not result in a significant increase in serum carboxypeptide type II collagen (CPII) nor in chondroitin sulfate-846 (CS-846), which are biomarkers released during an attempt at cartilage repair. However, concentrations of these markers demonstrated a tendency to increase when the duration and intensity of training increased [31].

Korac et al. [32] examined local and systemic effects of a specific PUFA-based supplement on horses challenged with equine interleukin-1β. The authors used the STRUCTURE-Joint (ST-J) supplement, designed to enhance the provision of omega-6 to omega-3 acids in horses’ diets. The horses received 120 mL of ST-J for 30 days. Supplementation significantly increased synovial levels of resolvin D1, a specialized mediator that helps to reduce inflammation. Increased synovial nitric oxide and PGE2 were observed, which the authors suggested might actually assist in tissue adaptation and blood flow during stress. The horses also showed a reduced joint circumference (less swelling) after the challenge. The authors concluded that the supplement promotes the resolution phase of inflammation rather than just blocking the initial response [32].

A summary of studies discussed above is presented in Table 1.

## 6. Fatty Acids Content in Mare Milk and Its Effect on Foal Development

Mother’s milk is the first food provided to all mammals, including horses. For this reason, its composition is a factor which may affect the health and development of offspring.

The study by Barłowska et al. [33] compared the nutritional and health-promoting properties of milk from two different horse breeds: the cold-blooded Sokólski and the warm-blooded Polish Halfbred. Focusing on the fatty acids profile, both breeds showed a very favorable ratio of unsaturated to saturated fatty acids. The milk from the Sokólski breed contained higher levels of saturated fatty acids (SFAs) and certain monounsaturated fats, while Polish Halfbred milk had a significantly higher concentration of polyunsaturated fatty acids (PUFAs), including the essential linoleic and α-linolenic acids. This gave Halfbred milk a slightly better profile regarding cardiovascular health indicators. Another study [34] aimed to characterize the nutritional value of Chilean Corralero milk over the first four months of lactation to see how time affects its quality. The authors observed that the milk was rich in polyunsaturated fatty acids (PUFAs) and had a low n-6/n-3 ratio (approximately 2.5:1), which is highly beneficial for reducing systemic inflammation. Moreover, milk of this breed contained significant amounts of linoleic acid and α-linolenic acid. The study conducted by Pikuł et al. [35] focused on how the stage of lactation, the age of the mare, and the number of foals influence the fat content and fatty acid (FA) profile of milk from Polish Konik horses. The study demonstrated that their milk is characterized by a high proportion of polyunsaturated fatty acids (PUFAs) and a very low level of trans-fatty acids. Konik horse milk is particularly rich in linoleic acid (C18:2) and α-linolenic acid (C18:3). The ratio of unsaturated to saturated fatty acids in Konik milk is much higher than in cow’s milk, making it more similar to human milk and highly desirable for cardiovascular health. Moreover, unlike ruminant milk (cow/sheep), mare milk contains very small amounts of short-chain fatty acids (C4:0 to C10:0). This is a key reason why mare milk has a much milder, less “animal” odor and flavor.

Boranbayeva et al. [36] investigated the milk of mares kept in traditional pasture-based systems, focusing on how age and the timing of foaling (season) affect the milk’s nutritional density. The study demonstrated that mares that foaled in the spring and grazed on lush, young pasture produced milk with significantly higher concentrations of unsaturated fatty acids compared to those foaling later. In addition, direct grazing significantly enriched the milk with α-linolenic acid (ALA), as the fresh forage provided a direct source of omega-3 precursors. Chen et al. [37] conducted a comparative study of the milk of cows, goats, camels, donkeys, and mares. Mare milk was found to have a ratio of saturated to unsaturated fatty acids (1:0.69:0.84) closest to that of the optimal human nutritional guidelines established by the FAO/WHO. Mare milk also contained the highest percentage of polyunsaturated fatty acids (PUFAs) among all species studied, far exceeding that of cows or goats. Gregić et al. [38] conducted a longitudinal study of the world-famous Lipizzaner breed, tracking how the fatty acid profile of their milk evolves from colostrum to late lactation (up to 150 days). Saturated fatty acids (SFAs) were highest in the colostrum but decreased as lactation progressed. Conversely, the “healthy” unsaturated fats remained more stable or improved in their ratio over time. Lipizzaner milk showed a high concentration of capric (C10:0) and lauric (C12:0) acids, which are known for their powerful antimicrobial and antiviral properties. The study calculated the “Atherogenic Index” (risk of heart disease) for the milk and found it consistently decreased as the mares moved further into lactation, making mature Lipizzaner milk exceptionally heart-healthy for consumers.

The above studies indicate that the fatty acid profile of mare’s milk depends on many factors, such as breed, stage of lactation, environmental factors, and nutrition. This profile may also change under the influence of supplementation, thus affecting development of offspring.

Recent studies have shed new light on so-called fetal programming. The review article by Chavatte-Palmer and Robles [39] explored the concept of Developmental Origins of Health and Disease (DOHaD) in horses. It examined how the nutritional and physiological environment provided by a mare during pregnancy “programs” the long-term health, metabolism, and athletic potential of a foal. The mechanism of programming includes environmental factors (like a mare’s diet), which can cause persistent modifications in gene expression in an offspring without changing the DNA sequence itself. Moreover, the placenta is a kind of master regulator. It senses the maternal environment and adapts its nutrient transport and blood flow to protect the fetus. However, these adaptations can lead to permanent changes in the foal’s organs and metabolic systems. The study concludes that mare nutrition is not just about producing a live foal, but about determining the quality of that foal’s life. Proper management of mares can reduce the incidence of orthopedic diseases and metabolic disorders in the next generation of equine athletes [39].

The study by Snyder-Peterson et al. [40] aimed to compare the efficiency of maternal supplementation with flax oil (high in ALA) versus fish oil (high in EPA/DHA) in altering foals’ fatty acid profiles. Mares were fed supplements starting at day 310 of gestation through early lactation. The authors observed that the foals born to mares supplemented with fish oil had significantly higher DHA levels in their plasma at birth compared to those in the flax group. The study suggested that horses (both mares and foals) are relatively inefficient at converting ALA (from flax) into the more beneficial DHA, making direct DHA/EPA supplementation (fish oil) more effective for increasing these levels in offspring. Moreover, the authors suggested that DHA consumed by mothers may be preferentially transferred to their foals during pregnancy [40].

Another study aimed to examine the effects of fish oil and fish/soy oil blends on milk IgG, placental efficiency, and fatty acid composition in both mares and foals [41]. The authors noted that supplementation did not increase the concentration of Immunoglobulin G (IgG) in the mare’s milk or the foal’s serum, meaning it did not directly enhance passive immunity transfer. There were no significant effects on insulin or glucose concentrations in either the mare or the foal. Maternal fat supplementation did not significantly alter placental weight or efficiency. The fatty acid profile of the milk was successfully altered to reflect the mare’s diet, providing more omega-3s to the nursing foal.

The research by Kouba et al. [42] evaluated how supplementing a mare’s diet with marine-derived omega-3 fatty acids, specifically DHA (docosahexaenoic acid) and EPA (eicosapentaenoic acid), affects the fatty acid profiles of her milk and her foal’s plasma, as well as her own reproductive recovery after foaling. The study was conducted on 20 pregnant mares. Starting 60 days before their expected foaling date, the mares were assigned to one of three diets: a control supplemented with corn oil, and two experimental groups, supplemented with a DHA-rich source (12.64 g/day) and with a blend of EPA and DHA (8.84 g EPA and 10.43 g DHA/day). Plasma was collected from the mares and foals; milk was collected at various stages of lactation. The researchers also monitored follicular growth and prostaglandin (PGFM) levels to track the first postpartum ovulation. The study demonstrated that the mares in the experimental groups had significantly higher concentrations of DHA and total n-3 fatty acids in their plasma and milk compared to the control group. The foals from the supplemented mares showed significantly higher plasma concentrations of DHA at birth and throughout the first 30 days of life, proving that maternal supplementation successfully transfers these beneficial fats to the offspring through the placenta and milk. Considering the mare reproductive variables, supplementation did not significantly affect the time it took for the mares to reach their first postpartum ovulation or the size of the pre-ovulatory follicle. While omega-3s are often thought to reduce inflammation, the study found no significant difference in the concentrations of PGFM (a marker of uterine involution) between the diet groups. The study also noted that while both supplements were effective, the EPA + DHA group had the highest total omega-3 concentrations in the milk, suggesting that a blend may provide a more robust fatty acid profile for nursing foals. The study concluded that supplementing mares with long-chain omega-3 fatty acids during late pregnancy and early lactation is an effective way to increase the omega-3 status of foals. However, at the doses provided, these supplements did not significantly alter the mare’s reproductive timeline or prostaglandin levels following birth [42].

## 7. Stallions Reproduction

A problem in stallions is the high variability in semen in terms of its suitability for frozen storage, which means that the use of frozen semen for artificial insemination is quite limited. Even for stallions considered to be good donors of semen that can be cryopreserved, a large number of ejaculates do not meet the requirements for sperm motility after thawing. Among other things, seasonal changes in the reproductive physiology of stallions contribute to this.

The study by Brinsko et al. [43] aimed to determine if supplementing the diet with docosahexaenoic acid (DHA) would improve the quality of fresh, cooled, and frozen semen. Eight stallions participated in a crossover study where they received either a standard diet or a diet supplemented with 250 g of a DHA-enriched nutraceutical for 14 weeks. The authors observed that supplementation resulted in a threefold increase in DHA levels in the semen. No significant changes in sperm motility were observed in the fresh semen, while in the cooled semen (after 48 h) there was a significant improvement in the total, progressive, and rapid motility. The greatest benefits were noted in stallions with lower fertility, whose semen typically responded poorly to the cooling and storage process. Supplementation also improved the sperm motion parameters after thawing (frozen semen) [43].

The study conducted by Schmid-Lausigk and Aurich [44] focused on counteracting the seasonal decline in the quality of frozen and cooled semen that occurs in stallions during late winter. For 84 days (from November to February), six stallions received 100 mL of linseed oil (LO) plus an antioxidant supplement (Vitamin E, Selenium, and SOD), while five stallions served as the control group. The study demonstrated that the quality of frozen and cooled semen naturally declined in all horses by February as compared to November. Supplementation with linseed oil and antioxidants mitigated the decline in motility and membrane integrity in the cooled-stored semen. However, the diet did not prevent the deterioration of semen quality after thawing during the late winter period. The supplemented stallions showed a more pronounced decrease in velocity parameters (VAP, VCL, and VSL) of frozen semen compared to the control group [44].

Both studies indicate that appropriate supplementation with omega-3 fatty acids (DHA or linseed oil) can benefit the preservation of stallion semen, particularly cooled semen. While DHA appears to help stallions with poor semen quality, the combination of linseed oil and antioxidants helps protect sperm from the negative effects of winter seasonality.

The study by Rodrigues et al. [45] evaluated the effect of PUFA supplementation in stallions’ diets on their semen quality (fresh, cooled and frozen–thawed). Stallions in the experimental group received an addition of 150 mL of linseed oil for 60 days. Semen samples were collected and their quality was evaluated. No effect of supplementation was found in the fresh or cooled semen, but the quality of frozen–thawed semen improved in the supplemented stallions. Parameters like vigor, viability, osmotic stress tolerance, acrosome integrity and thiobarbituric acid reactive substances (TRARs) were highest in the experimental group, indicating that adding omega-3 FAs to stallions’ diets may be a good option to improve frozen–thawed semen quality, increasing its resistance to osmotic changes during freezing [45]. A summary of studies discussed above is presented in Table 2.

## 8. Possible Mechanisms of Omega-3 Fatty Acids Action in Supplemented Horses’ Diets

Possible mechanism of effects observed as a result of supplementing horses’ diets with omega-3 fatty acids are summarized in Table 3. They mostly emphasize the role of omega-3 fatty acids in inflammation reduction, as well as the competitive nature of omega-6 and omega-3 acids.

It is worth bearing in mind that most of the suggested mechanisms of action for omega-3 fatty acids are derived from studies on other species, not on horses. Furthermore, it should be emphasized that omega-3 fatty acids should be regarded as supportive nutritional interventions rather than therapeutic agents.

## 9. Limitations, Knowledge Gaps and Future Research

There are certain limitations to the research on the use of omega-3 fatty acids supplements in horses. The main problem with horses is usually the small sample size, as well as the lack of animal standardization, in terms of age, sex or general condition. Another problem may be seasonal changes in basic forage quality, which may affect study results. Thus, stable feed and environment should be taken into account when planning future studies.

Comparison of various studies may be also hindered by variable study protocols or heterogenous outcome measures. Another issue that should be mentioned is the fact that mechanisms of omega-3 fatty acids action in the case of horse supplementation are mostly extrapolated from other species.

Therefore, future research should focus on attempting to elucidate these mechanisms directly in horses. Moreover, the mechanisms underlying the relationships between inflammation, insulin dysregulation and laminitis need to be understood for studies related to equine metabolic syndrome. Similarly, such relationships should be examined in cases of other conditions, as it seems that inflammation is the common thread running through all the research.

In addition, future trials evaluating respiratory and metabolic improvements should integrate a comprehensive analysis of oxidative stress markers to definitively validate how fatty acids reduce tissue damage. Research is also needed to isolate the precise therapeutic dosing brackets for specific conditions to prevent over-supplementation beyond the plasma integration limit. Studies should continue to prioritize and fine-tune marine-derived (algae/fish) options over plant-derived options (flax), given horses’ proven inability to efficiently convert ALA into long-chain DHA/EPA.

Given that maternal nutrition during pregnancy persistently modifies gene expression and organ development in offspring via the placenta, extended multi-year studies are needed to evaluate the explicit athletic and metabolic outcomes of adult horses whose mothers were supplemented with omega-3 fatty acids.

Future research should also examine the efficacy of combining omega-3 fatty acids with other supportive functional compounds in addition to studying them exclusively in isolation.

## 10. Conclusions

Omega-3 fatty acids, particularly EPA and DHA, play a significant role in equine diet supplementation. The evidence indicates that their anti-inflammatory, metabolic, and structural benefits may support various conditions’ treatment, and may be a valuable addition to the diet. It should be emphasized that marine-derived sources are more effective than plant-based precursors due to the limited endogenous conversion of ALA to long-chain derivatives. However, the presented narrative review also reveals some limitations; thus, further research is needed to recognize the mechanisms of omega-3 fatty acids’ actions as a feed supplement in a more complex and comprehensive manner.

## Figures and Tables

**Table 1:** Summary of the scheme and main findings of the analyzed dietary interventions concerning airway diseases, equine metabolic syndrome, and joint and musculoskeletal system disorders.

Study	Number of Animals	Omega-3 Source	Dose	Duration	Investigated Condition	Main Findings
Nogradi et al. [11]	32	omega-3 PUFA supplement (no details provided)	30 g/d (DHA dose 2.5–3.8 mg/kg BW) or 60 g/d (DHA dose 5.1–9.1 mg/kg)	8 weeks	recurrent airway obstruction (RAO) and inflammatory airway disease (IAD)	greater improvement in clinical signs (cough score, respiratory effort, BALF neutrophils) in supplemented horses
Khol-Parisini et al. [14]	9	seal blubber oil	320 mg/kg BW/d (70–75 mg/kg BW/d of omega-3 PUFAs)	10 weeks	recurrent airway obstruction (RAO)	omega-6 to omega-3 ratio decrease in blood plasma and leukocytes; reduction in pulmonary epithelial lining fluid (PELF) leukocytes count
Elzinga et al. [17]	10	DHA-rich microalgae	16 g DHA/d	46 days	equine metabolic syndrome	no effect on insulin sensitivity; inflammatory cytokine measures decrease in supplemented group in relation to basal measurements (not to the control)
Hess et al. [24]	21	fish and algae supplement, flaxseed meal	38 g of omega-3 fatty acids	90 days	insulin sensitivity	no effect on insulin sensitivity, β pancreatic response or glucose-mediated glucose disposal
Loos et al. [26]	16	pelleted supplement containing blend of marine-derived omega-3 fatty acid sources	0.75 g of supplement/kg BW/d	4 weeks	insulin dysregulation	reduced progression of insulin resistance in predisposed horses
Manhart et al. [29]	16	pelleted omega-3 supplement	300 g/d (15 g of EPA, 19.8 g of DHA)	90 days	arthritis	omega-6 to omega-3 ratio decrease in blood plasma; decrease in synovial fluid white blood cells count; lower plasma prostaglandin E2 and fibrinogen levels
Ross-Jones et al. [30]	12	marine-derived omega-3 supplement	40 g of omega-3 PUFAs/d	91 days	experimentally induced synovitis	EPA and DHA content increase in serum and synovial fluid; decrease in ADAMTS-4 expression; no effect on synovial fluid’s PGE2 content or MMP activity
Leatherwood et al. [31]	16	marine-derived omega-3 supplement	700 g/d (15 g of EPA and 20 g of DHA)	140 days	markers of joint inflammation during training	plasma EPA and DHA levels increase; no effect on serum carboxypeptide type II collagen (CPII) or chondroitin sulfate-846 (CS-846)
Korac et al. [32]	16	STRUCTURE-Joint PUFA supplement	120 mL/d	30 days	intra-articular challenge with recombinant equine interleukin 1β (IL-1)	nitric oxide, resolvin D1 and PGE2 increase in synovial fluid; joint circumference decrease

**Table 2:** Summary of the scheme and main findings of the analyzed dietary interventions concerning foal development and stallions’ semen characteristics.

Study	Animals	Omega-3 Source	Dose	Duration	Samples Examined	Main Findings
Snyder-Peterson et al. [40]	13 mares and their foals	fish oil, flaxseed	242.4 g of fish oil and 90 g of flaxseed per day (40 g of omega-3 acids/d)	day 310 of gestation to day 5 post-parturition	mare milk samples; mare and foal plasma samples	mean plasma DHA level higher in foals born to flaxseed-supplemented mares
Hodge et al. [41]	18 mares and their foals	fish oil; blend of fish oil and soybean oil	0.11 mL of fish oil/kg BW; 0.33 mL of fish and soybean oil blend/kg BW	28 days before parturition to day 84 after foaling	blood samples from mares and foals; colostrum, milk, placenta	no effect on mare or foals’ plasma IgG, serum insulin or plasma glucose; changes in omega-3 FAs content in milk
Kouba et al. [42]	20 mares and their foals	corn oil, DHA, EPA + DHA from fish oil or algae	113.4 of corn oil/d; 12.64 g DHA/d; 8.84 g EPA + 10.43 g DHA/d	60 days before parturition to 2nd postpartum estrus	mare milk samples; mare and foal blood samples	higher DHA and total omega-3 concentrations in plasma and milk of supplemented mares; higher plasma DHA in foals from supplemented mares
Brinsko et al. [43]	8 stallions	fish oil-based nutraceutical	250 g/d (25% DHA, 5% EPA, min. 34% omega-3 FAs)	14 weeks	semen samples	increased sperm DHA level; no effect on motion features in fresh semen; improved motility in cooled semen
Schmid-Lausigk and Aurich [44]	11 stallions	linseed oil	100 mL/d (60% α-linolenic acid, 15% linoleic acid)	84 days	semen samples	attenuation of seasonal semen quality decrease (motility, membrane integrity)
Rodrigues et al. [45]	10 stallions	linseed oil	150 mL/d	60 days	semen samples	no effect on fresh or cooled semen; improved quality of frozen–thawed semen

**Table 3:** Possible mechanisms of omega-3 fatty acids actions in analyzed conditions.

Condition	Proposed Mechanisms	References
Airway inflammatory diseases	Competitive replacement of some of the arachidonic acid in leukocyte cell membranes resulting in anti-inflammatory effects. Change in the conversion of arachidonic acid towards less inflammatory compounds. Shift in the balance towards omega-3 metabolic pathways. Increased production of 3-series prostaglandins and 5-series leukotrienes. Reduced chemotaxis or reduced synthesis of eicosanoids with chemotactic properties.	[2,11,13,14]
Equine metabolic syndrome	Improvement in insulin sensitivity, which presumably modulates concentrations of circulating fatty acids, resulting in inflammation decrease. Enhanced production of anti-inflammatory resolvins and protectins. Activation of peroxisome proliferator-activated receptor gamma (PPAR-γ). Inhibition of nuclear factor kappa B (NF-κB) pathway. Increased cell membranes’ fluidity, improving glucose transport.	[16,17,19,20,23,26]
Joint inflammation and musculoskeletal system disorders	Replacement of arachidonic acid by omega-3 acids in cell membranes, leading to production of less pro-inflammatory compounds. Reduced production of pro-inflammatory eicosanoids. Shift in joint environment balance, from catabolic to anabolic. Change in the composition of synovial fluid and joint tissues.	[29,30,31,32]
Foal development	Transfer of omega-3 fatty acids from mother to foal through placenta and milk. Increased concentrations of omega-3 acids, especially DHA, in foal tissues, potentially improving their development and cognitive functions.	[40,41,42]
Stallion sperm quality	Direct incorporation of DHA into sperm phospholipid bilayer. Shifting the balance between omega-6 and omega-3 fatty acids in sperm cells. Increase in membrane fluidity and permeability. Cold shock protection due to increased membrane fluidity. Possible involvement in sperm maturation.	[43,44,45]

## Data Availability

No new data were created or analyzed in this study. Data sharing is not applicable to this article.
